# Development of a cell-seeded modified small intestinal submucosa for urethroplasty

**DOI:** 10.1016/j.heliyon.2016.e00087

**Published:** 2016-03-10

**Authors:** Long Zhang, Anna Du, Junping Li, Minjie Pan, Weiwei Han, Yajun Xiao

**Affiliations:** a Department of Urology, Union Hospital, Tongji Medical College, Huazhong University of Science and Technology, Wuhan, Hubei, China; b The Core Facility and Technical Support, Wuhan Institute of Virology, Hubei, China

**Keywords:** Regenerative medicine, Biomaterials, Plastic surgery

## Abstract

**Objective:**

To explore the feasibility of a modified 3D porous small intestinal submucosa (SIS) scaffold seeded with urothelial cells (UC) for surgical reconstruction in a rabbit model.

**Material and methods:**

Eighteen New England white male rabbits were divided into three groups and a 0.8 × 1.5 cm^2^ section of the anterior urethral mucosa was removed from each animal. Ventral onlay urethroplasty was performed with a 1.0 × 1.7 cm^2^ SIS scaffold that was either cell-seeded and treated with 5% peracetic acid (PAA) (n = 6), or cell-seeded and untreated (n = 6), or unseeded and treated with 5% PAA (n = 6). Animals were sacrificed at 6 months post-repair and retrograde urethrography and histological analyses performed.

**Results:**

In animals implanted with cell-seeded and PAA treated SIS scaffolds, urethrography showed wide-caliber urethra without any signs of stricture or fistulae, and histological analyses confirmed a complete urethral structure. In contrast, ulceration and fistula occurred in the reconstructed urethra of animals implanted with cell-seeded but untreated SIS scaffolds, and evident stricture was present in the unseeded, PAA treated group. Histological analyses demonstrated less urothelial coverage and smooth muscle in the cell-seeded and untreated SIS scaffold group, and serious fibrosis formation occurred in the unseeded, treated group.

**Conclusions:**

A modified 3D porous SIS scaffold seeded with UC and treated with PAA produces better urethroplasty results than cell-seeded untreated SIS scaffolds, or unseeded PAA treated SIS scaffolds.

## 1 Introduction

A variety of congenital and acquired urethral pathologies including hypospadias, stricture, fistulae and straddle injuries can severely impair its normal function, necessitating surgical reconstruction [1, 2, 3, 4]. Penile skin and oral mucosa are often used to address long urethral defect [5, 6, 7]. However, in addition to the potential donor site morbidity, there is a lack of adequate amount of those grafts in many cases, then urothelial cell based tissue engineering has shown promise as alternative for urethral substitution [8, 9, 10, 11, 12].

Dorin et al. demonstrated that the maximum distance suitable for normal tissue formation over a defect, using an unseeded cellular graft, which depends on native tissue regeneration, appears to be less than 1 cm; whereas the repair of larger defects (1 cm or more) appears to require cell seeded grafts for higher success rates [13]. Xie et al. used stretched electron-spun silk fibroin matrix to provide 3D porous scaffold seeded with urothelial cells for urethra reconstruction. However, silk fibroin did not contain as many structural elements for regeneration, such as collagen, elastin, dermatan sulfate, heparin and growth factors, as natural collagen matrix [10]. Despite the fact that those molecules are adequate in natural collagen matrix, the high density and retained heterogeneous cellular compounds limit its use [14].

Recently, it was reported that a modified 3D porous natural collagen matrix, such as SIS and bladder acellular matrix (BAM), after decellularization and oxidation with 5% peracetic acid (PAA) was free of cellular compounds, showed highly porous microstructure, promoted the formation of a multilayer of transitional epithelium structure *in vitro* and led to better *in vivo* tissue regeneration in a nude mice model [15, 16]. Such modified SIS may be a kind of new ideal material seeded with cells for reconstruction of long urethral defect (1 cm or more), which is one of the most complicated issues in clinical practices. In this study, we further investigated the urethral tissue regeneration using such modified 3D porous SIS scaffold (1.7 cm) seeded with UC in a rabbit model of large urethral mucosa defect (1.5 cm).

## 2 Material and methods

This study was conducted with the approval of the Institutional Animal Ethics Committee. A total of 18 New England white male rabbits were divided into 3 groups: cell-seeded 5% PAA treated SIS (n = 6), cell-seeded 0% PAA treated SIS (n = 6), unseeded PAA treated SIS (n = 6). Constructs were evaluated at 6 months after urethral surgery.

### 2.1 Preparation of the modified 3D porous SIS scaffold

**Fig. 1:**
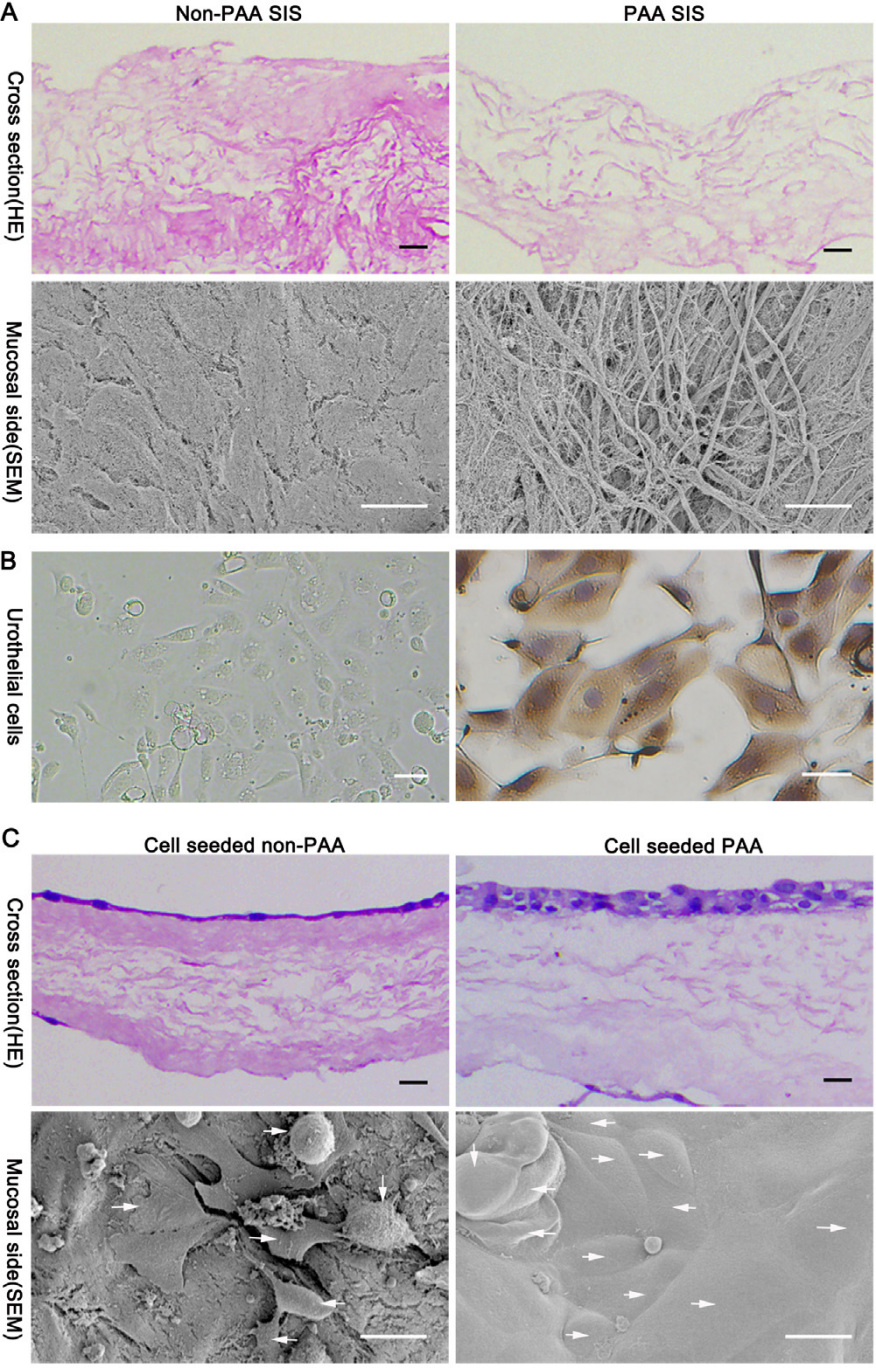
(A) Cross section of acellular small intestinal submucosa (SIS) with 0% PAA treatment (Upper, Left) and SIS with 5% PAA oxidation (Upper, Right), HE = Hematoxylin and eosin, scale bar = 20 μm; Mucosal side of 5% PAA treated SIS (Lower, Right) seemed to be more porous compared to non-PAA treated SIS (Lower, Left), SEM = Scanning Electron Microscope, scale bar = 10 μm, (B) primary culture of bladder urothelial cells (Left), scale bar = 40 μm; cells showed expression of AE1/AE3 (Right), scale bar = 40 μm, (C) bladder urothelial cells formed a slender layer on decellularized SIS without oxidation treatment (Upper, Left), cells formed multiple layers on the mucosal side of the modified 3D porous matrix (Upper, Right), HE, scale bar = 20 μm; SEM showed cells were rounded or polygonal and attached to non PAA SIS with pseudopodial stretching peripherally (Lower, Left), cells were cuboid or ellipsoid and more densely populated on 5% PAA treated SIS (Lower, Right), scale bar = 15 μm, arrowheads indicate the UC.

The procedure was conducted as reported previously [15]. Briefly, the mucosa of the fresh porcine intestine was manually removed and rinsed with distilled water in a stirring flask at 200 rpm and 4 °C for 2 days, followed by treatment with peracetic acid (PAA, XiLONG SCIENTIFIC, Shanghai, China) at a concentration of 0 or 5% (v/v) for 4 h. This matrix was sequentially treated with 1% Triton X–100 solution for 2 days and then rinsed with distilled water for additional 2 days. Finally, the SIS scaffold was sterilized using 0.1% PAA in 20% alcohol for 2 h, washed 3 times with sterilized distilled water for 10 min each and stored in sterilized distilled water at 4 °C until further use (Fig. 1).

### 2.2 Histological analyses and scanning electron microscopy (SEM)

The PAA treated and non-PAA treated SIS were fixed in 4% paraformaldehyde at room temperature for 12 h, embedded in paraffin and then sectioned for Hematoxylin and eosin (HE) to detect the cellular materials. The SIS scaffolds were fixed in 2.5% glutaraldehyde at 4 °C for 12 h. The scaffolds were dehydrated with graded ethanol, and dried. The mucosal side image was obtained at 3.0 kV, 800 × magnification using scanning electron microscope (SU8010, HITACHI, Japan).

### 2.3 Bladder biopsy

A bladder biopsy was obtained from 12 cell-seeded SIS group rabbits. The animals were anesthetized using 1 ml/kg 3% pentobarbital (Merck, Darmstadt, Germany). After surgical preparation, a 3-cm median incision was made starting 0.5 cm above the pubic bone, through which the bladder was exposed, a 0.6 × 0.6 cm^2^ bladder wall was sampled. The bladder, abdominal muscle and skin were then sutured in layers. An intramuscular injection of 6 mg/Kg Enrofloxacin (Baytril^®^, Kiel, Germany) was given to each rabbit before anesthesia and 3 days post-surgery.

### 2.4 Cell isolation and expansion

The samples were digested in 3 ml 1% (w/v) pronase E (Sigma-Aldrich, Saint Louis, MO) solution containing 0.01 M sodium acetate and 0.005 M calcium acetate at 4 °C overnight. Only UC were obtained by gentle scraping from the digested mucosal surface of the sample and expanded in Epithelial Cell Medium (Sciencell, San Diego, CA) supplemented with 5% (v/v) fetal bovine serum (FBS), 1% penicillin/streptomycin and 1% (v/v) epithelial growth factor (EGF). Culture medium was changed 3 times per week. Passage 2 of the primary cells was obtained for integration into the graft. Prior to seeding, UC were cultured on coverslips, fixed by 4% paraformaldehyde, rinsed with PBS, and immunostained using monoclonal AE1/AE3 antibody (Abcam, Cambridge, MA) to detect pancytokeratins.

### 2.5 Cell-seeded SIS *in vitro*

*Ex vivo* expanded UC were seeded on the luminal side of the preconfigured sterile 0% or 5% PAA treated SIS scaffolds at a concentration of 2 × 10^6^/cm^2^. Cells were allowed to attach to the matrix for 24 h. The cell-seeded compounds were then cultured for 2 weeks with 3 media changes per week. The cell-seeded compounds samples were also fixed for HE and SEM (3kv, 400 × magnification) as mentioned above.

### 2.6 Surgical procedure

**Fig. 2:**
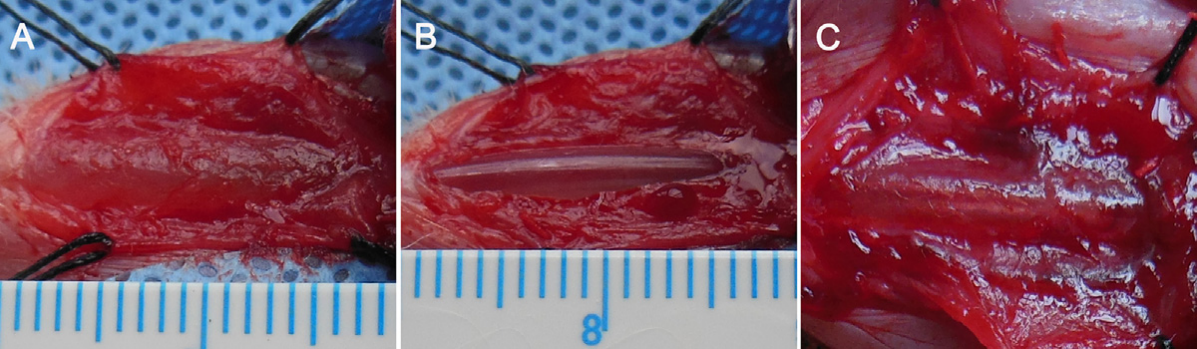
(A) Exposure of penile urethral mucosa, (B) excision of urethral mucosa (0.8 × 1.5 cm^2^, width × length), (C), cell-seeded SIS graft (1.0 × 1.7 cm^2^) was sutured on the urethral defect.

After anesthesia of pentobarbital, the urethra was catheterized and exposed through a ventral middle line incision of skin and corpora spongiosum, a 0.8 × 1.5 cm^2^ (width × length) ventral penile urethral mucosa defect was created under 2.5 × optical magnification, starting 1 cm proximal to the external urethral meatus. A biomaterial of 1.0 × 1.7 cm^2^ was anastomosed to the defect site using running 8–0 coated VICRYL™ suture. The wounds were closed with interrupted suture in layers [17] (Fig. 2).

### 2.7 Follow-up

The urethral stent was left in place for 2 weeks. Animals were submitted to retrograde urethrography and euthanized at 6 months post-repair. After sacrifice, the urethra was removed for gross examination and histological processing. All tissues were fixed in 4% paraformaldehyde at room temperature for 12 h, embedded in paraffin and then sectioned for Hematoxylin and eosin (HE) and Masson’s trichrome (MTS) were performed. Immunohistochemical (IHC) analyses were conducted using monoclonal pancytokeratins AE1/AE3 antibody (Abcam, Cambridge, MA) to detect UC, anti-α-SMA antibody (Abcam) to detect smooth muscle, anti-CD31 (Abcam) to detect endothelial cells. Histomorphometric analyses were performed to evaluate the degree of epithelial tissue regeneration using Image J software v1.47 (NIH, Bethesda, MD). All measurements were performed based on 8 to 12 independent microscopic field dispersed equally along distal, proximal and central region of the engineered urethra. The relative content of epithelium, smooth muscle and vessel were quantified as the percentage of AE1/AE3+ area, α-SMA+ area and CD31+ vessel area in total area examined, respectively.

### 2.8 Statistical analyses

Data were expressed as mean ± SD, and analyzed with analysis of variance (ANOVA) to determine the difference among 3 groups using computer software (SPSS 19.0, Chicago, IL). A *p* value < 0.05 was considered significant.

## 3 Results

### 3.1 *In vitro* analyses

A cross section of 5% PAA treated SIS (Fig. 1A, Upper, Right) seemed more porous and to be decellularized more completely compared to 0% PAA treated SIS (Fig. 1A, Upper, Left). Scanning electron microscope (SEM) showed the mucosal side of 5% PAA treated SIS (Fig. 1A, Lower, Right) more porous than non-PAA SIS (Fig. 1A, Lower, Left). UC were grown and expanded until the appropriate numbers of cells were reached. UC showed a typical cobblestone appearance under the inverted microscope (Fig. 1B, Left). UC showed expression of AE1/AE3 (Fig. 1B, Right). The seeded UC formed 2 ∼ 3 layers of transitional epithelium on the luminal side of the 5% PAA SIS scaffold (Fig. 1C, Upper, Right). However, single layer of UC was found on 0% PAA SIS scaffolds (Fig. 1C, Upper, Left). SEM demonstrated that cells were populated more densely on PAA treated SIS, UC proliferated rapidly, fused, overlapped, had the shapes of ellipse or polygons and connected with each other tightly to become multi-layered structure (Fig. 1C, Lower, Right). By comparison, UC on non-PAA treated SIS formed pseudopodia and extended peripherally but could not fuse to become complete lamellar, and the non-PAA treated SIS was still seen between UC (Fig. 1C, Lower, Left).

### 3.2 Surgical outcomes

**Fig. 3:**
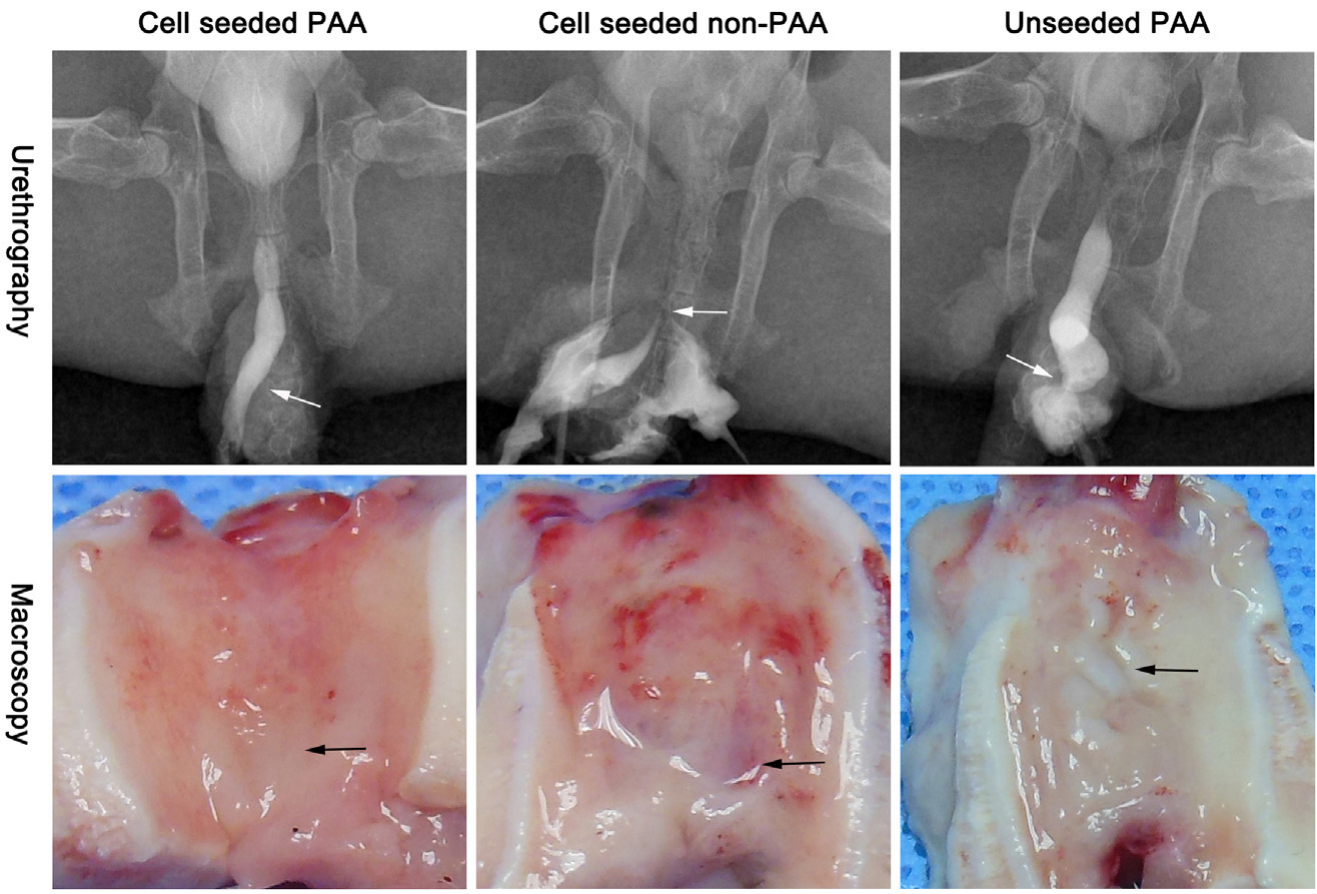
Retrograde urethrography and macroscopic anatomical views 6 months after surgery showed patent urethra (Upper, Left) and normal-like mucosa (Lower, Left) in animals implanted with cell-seeded 5% PAA treated SIS scaffolds; irregular mucosa, ulceration (Lower, Middle) and fistula (Upper, Middle) developed in cell-seeded non-PAA group; stricture (Upper, Right) and scar/shrinkage (Lower, Right) were demonstrated in unseeded PAA group, arrowheads indicate the tissue engineered urethra.

The overall retrograde urethrography and macroscopy results are presented in Table 1. Animals implanted with cell-seeded 5% PAA-treated SIS scaffolds showed patent urethra with wide caliber without any sign of stricture or fistulae at 6 months post-repair in the retrograde urethrography (Fig. 3, Upper, Left), and urethra demonstrated normal-appearing mucosa without any ulceration, fibrosis or shrinkage under macroscopy (Fig. 3, Lower, Left). Irregular mucosa and ulceration developed in animals implanted with cell-seeded 0% PAA SIS scaffolds under macroscopy (Fig. 3, Lower, Middle), and urethrocutaneous fistulae was demonstrated in 3 of them in the retrograde urethrography (Fig. 3, Upper, Middle), the reconstructed urethra in unseeded PAA group was accompanied by stricture as evidenced by retrograde urethrography (Fig. 3, Upper, Right), and grossly, the urethral mucosa touched stiff and seemed pallid because of fibrosis formation and scarcity of vessel in sub-epithelial layer (Fig. 3, Lower, Right).

**Fig. 4:**
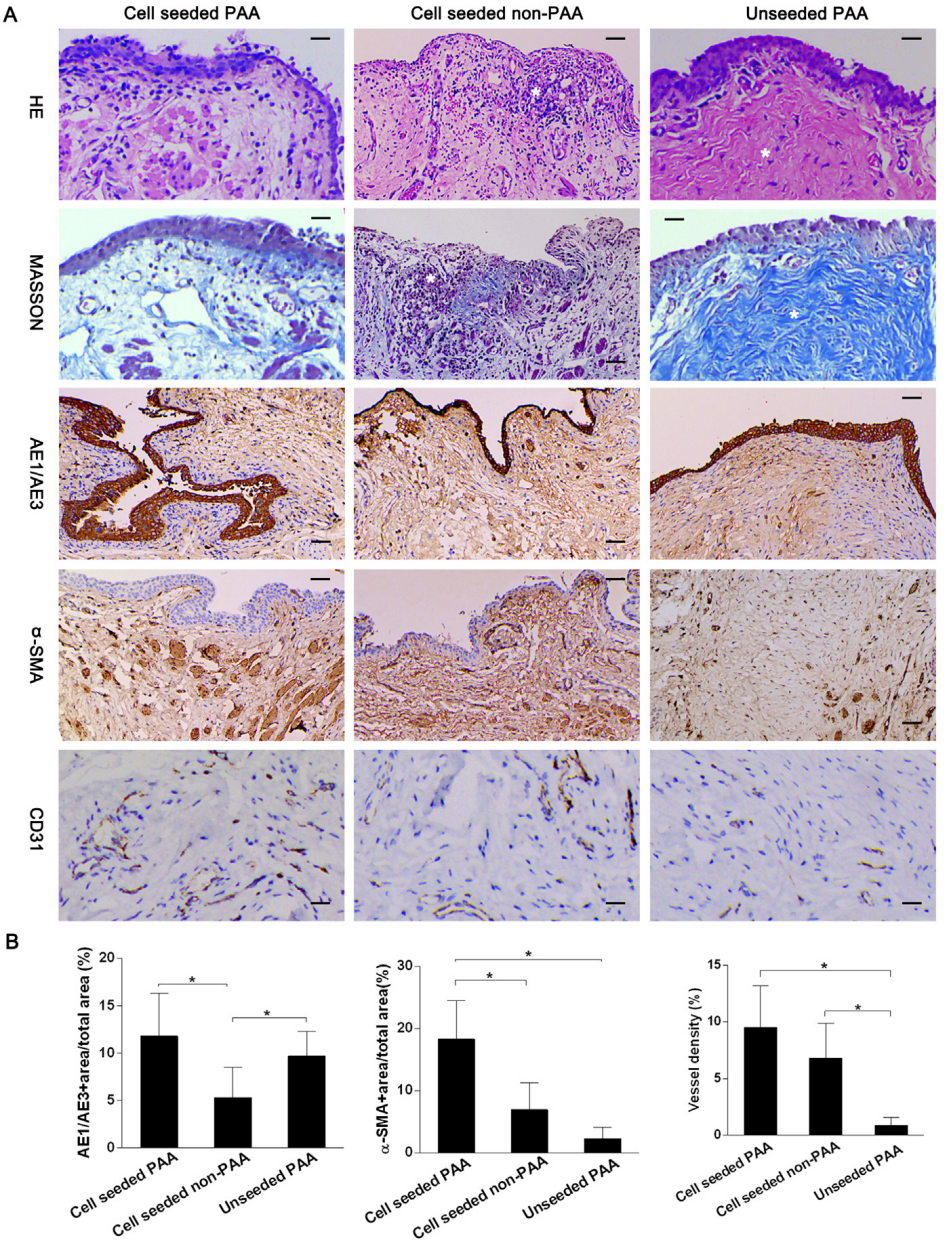
(A) Histological and immunohistochemistry analyses of reconstructed urethras. Animals implanted with 5% PAA treated cell-seeded scaffold showed formation of complete layers of transitional epithelium, more smooth muscle and vessels, scale bar = 20 μm; mononuclear cells aggregates in cell-seeded non-PAA group (*) scale bar = 20 μm; evident fibrosis formation (*) in unseeded PAA group, scale bar = 20 μm, (B) histomorphometric analyses of epithelium, smooth muscle and vessel among 3 groups (* *p* < 0.05).

Histological and immunohistochemistry analyses showed the reconstructed urethra had approximately normal epithelium, smooth muscle and vessels in animals treated with cell-seeded 5% PAA SIS. In contrast, less epithelia, smooth muscle and vessels were seen in animals treated with cell-seeded 0% PAA SIS scaffolds and in unseeded PAA groups. Mononuclear cells aggregates was shown in cell-seeded non-PAA group (*) and evident fibrosis formation (*) in unseeded PAA group (Fig. 4A). The average CK+ area in cell-seeded 5% PAA SIS group was significantly larger than in cell-seeded non-PAA group (*p* < 0.05). Animals in cell-seeded 5% PAA SIS group expressed significantly more α-SMA+ area than other two groups (*p* < 0.05). Animals implanted with cell-seeded PAA had significantly higher vessel density compared with unseeded PAA group (*p* < 0.05) (Fig. 4B).

## 4 Discussion

A variety of urethral congenital disorders as well as acquired pathologies can compromise its function. An end-to-end anastomosis can be used to repair short, non-complex defect. For long defects, skin or oral mucosa is often used for substitution. However, there is a lack of adequate amount of such grafts in many cases. Then tissue engineered urethral mucosa may be a promising alternative for the replacement.

Synthetic biomaterials such as PGA or PLGA and natural collagen-based materials including SIS and bladder acellular matrix (BAM) are mostly used scaffolds for urethral tissue engineering [18, 19, 20, 21]. The latter may allow tissue regeneration across the scaffold to proceed more quickly because of appropriate biological active molecules in them. However, the high density of collagen and retained heterogenic cellular materials are their two main disadvantages [14].

Most importantly, a scaffold with high porosity promotes cell proliferation and migration, and seems to allow more cells loading on the scaffold, thereby improving tissue regeneration and wound healing *in vivo* [22, 23, 24]. Recently, it was reported that treatment with 5% PAA lead to high porosity of SIS and BAM, almost completion of cellular compounds and still maintained 75% of their normal tensile strength. The mechanism may be related to its oxidation [15, 16]. In addition, we also found that many bubbles were produced following PAA treatment and led to the internal expansion of the SIS, which might also increase its porosity. The *in vitro* outcomes of the matrix in our study are consistent with theirs. The seeded cells proliferated and formed multiple uniform layer on 5% PAA treated SIS scaffold, which is necessary to keep water tight separation between periurethral and lumen in order to prevent urine leakage into the surrounding tissue and subsequent inflammation.

We further investigated the tissue regeneration supported by such modified 3-D porous SIS seeded with UC in a rabbit model of the ventral onlay urethroplasty. A 0.8 × 1.5 cm^2^ (width × length) of penile ventral mucosa was removed, followed by the replacement using a cell-seeded compound of 1.0 × 1.7 cm^2^. The application of a larger graft was chosen to decrease tensile stress on its anastomoses and graft itself. The surgical outcomes in our study are of importance, because defect of 1 cm or more has been shown to be too large to allow spontaneous tissue regeneration from the edge of anatomoses and to prevent fibrosis formation [13].

Animals receiving cell-seeded 5% PAA SIS graft showed improvement over other groups in both histological and functional aspects. Thin unorganized epithelial coverage, inadequate smooth muscle and urethra-cutaneous fistulae developed in cell-seeded 0% PAA SIS group at 6 months post-repair, chronic inflammation was also evidenced by mononuclear cell aggregates. Animals in unseeded PAA group showed serious stricture by retrograde urethrography and extensive fibrosis formation by histological assessment.

Fig. 3 provides us with representative characteristics of the individual group. Urethrography can demonstrate the wide caliber (Left), fistulae (Middle) or stricture (Right), as indicated by the arrowheads, of the regenerated urethra. The macroscopy can demonstrate the gross pathology change of mucosa, including normal-like (Left), ulceration (Middle), or scar/shrinkage (Right). Epithelium, sub-epithelial smooth muscle, vessels (Fig. 4A, AE1/AE3, α-SMA, CD31 staining, Left) regenerated very well in cell-seeded PAA group, so wide caliber was demonstrated in urethrogram (Fig. 3, Upper, Left) and normal-like mucosa under gross macroscopy (Fig. 3, Lower, Left).

The non-PAA matrix was less likely to prompt cell proliferation because of the disadvantages of high density and existence of retained heterogeneous cellular compounds, the epithelium in cell-seeded non-PAA is very thin (Fig. 4 AE1/AE3, Middle), and chronic inflammation (Fig. 4, HE/MASSON, Middle), ulceration (Fig. 3, Lower, Middle) occurred following the implantation under gross macroscopy, and fistulae formed in urethrogram (Fig. 3, Upper, Middle).

As a result of the scarcity of seeded UC in the unseeded group, the new epithelium regenerated from the native tissue is not solid enough to prevent the sub-epithelium from the urine leakage. So the inflammation and fibrosis formation occurred very early and seriously postoperatively. The mucosa with scar and shrinkage (Fig. 3, Lower, Right) touched stiff because of fibrosis formation in the sub-epithelium (Fig. 4, MASSON, Right), the caliber got stricture in urethrogram (Fig. 3, Upper, Right) and this kind of mucosa also seems pallid (Fig. 3, Lower, Right) because of scarcity of vessels in the sub-epithelium (Fig. 4, CD 31 staining, Right).

## 5 Conclusions

The results in the study detailed the feasibility of a modified 3-D porous SIS scaffold seeded with UC to serve as graft for onlay urethroplasty to treat large urethral mucosa defect. In comparison to cell-seeded non-PAA SIS scaffold and unseeded PAA SIS, 5% PAA SIS seeded with UC displayed better maintenance of the urethral patency, epithelization, smooth muscle proliferation and neovascularization. Future studies with bigger sample size are warranted to ascertain the potential of cell-seeded 5% PAA modified 3-D porous SIS graft for urethral reconstruction.

## Declarations

### Author contribution statement

Long Zhang: Conceived and designed the experiments; Performed the experiments.

Junping Li: Performed the experiments.

Anna Du, Minjie Pan, Weiwei Han: Performed the experiments; Contributed reagents, materials, analysis tools or data.

Yajun Xiao: Conceived and designed the experiments; Analyzed and interpreted the data; Wrote the paper.

### Funding statement

This work was supported by Innovation Fund of Wuhan Bureau of Human Resource and Social Security (85262).

### Competing interest statement

The authors declare no conflict of interest.

### Additional information

No additional information is available for this paper.

## Figures and Tables

**Table 1:** Overall urethrography and macroscopy results.

	Cell-seeded PAA	Cell-seeded non-PAA	Unseeded PAA
No. of rabbits	6	6	6
Urethrography (n)			
Wide caliber	6	3	0
Fistulae	0	3	0
Stricture	0	0	6
Macroscopy (n)			
Normal-like	6	3	0
Ulceration	0	3	0
Scar/shrinkage	0	0	6
